# Maternal exposure to a radio programme and maternal and child nutrition-related practices: cross-sectional analyses of the 2022 Nepal Demographic and Health Survey

**DOI:** 10.1017/jns.2025.10048

**Published:** 2025-10-30

**Authors:** Ramesh Prasad Adhikari, Subir K. Kole, Pooja Pandey Rana, Indra D. Kshetri, Kenda Cunningham

**Affiliations:** 1 Hellen Keller International Nepal, Green Block, Chakupat, Patan Lalitpur, Nepal; 2 Helen Keller International, New York, USA

**Keywords:** *Bhanchhin Amma*, Nepal, nutrition, radio, social and behaviour change communication

## Abstract

This paper examines associations between maternal exposure to a radio programme, *Bhanchhin Aama* (Mother Knows Best), and the programme’s most promoted maternal and child nutrition-related practices, using the Nepal Demographic and Health Survey (NDHS) from 2022. We limited our sample to mothers of children less than 2 years (*n* = 1,933). The primary exposure variable was whether the mother listened to the *Bhanchhin Aama* radio programme. The five primary outcomes were: maternal dietary diversity, maternal use of modern family planning methods, exclusive breastfeeding (EBF) of children less than 6 months, dietary diversity among children 6 to 24 months, and participation in growth monitoring and promotion among children 0 to 24 months. Descriptive analyses followed by logistic regression models, adjusted for potentially confounding factors and clustering, were conducted. Maternal exposure to *Bhanchhin Aama* was associated with nearly 70% higher odds of meeting both maternal (OR: 1.67; *p*: <0.001; CI: 1.26–2.21) and child minimum dietary diversity (OR: 1.70; *p*: 0.005; CI: 1.18–2.45), as well as 83% higher odds of a child participating in growth monitoring and promotion (OR: 1.83; *p*: 0.001; CI: 1.28–2.63). No associations were found for use of modern family planning methods and EBF. These findings suggests that radio programmes may be an effective tool to improve some maternal and child nutrition-related practices. Further research is needed to understand why certain behaviours are modifiable from this type of intervention versus others that are not and for which population groups this intervention would be most effective.

## Background

In 2012, the 65^th^ World Health Assembly (WHA) endorsed a comprehensive nutrition plan for mothers and children and outlined six global targets for nutrition to be met by 2025: reduce stunting among children under 5 years, reduce anaemia in women of reproductive age (15–49 years), reduce low birth weight, halt overweight and obesity among children under 5 years, increase exclusive breastfeeding (EBF) among children under 6 months, and reduce wasting in children under five.^(1)^ Despite continuous efforts to reduce maternal and child malnutrition,^(2)^ no country is on track to meet these targets.^(3,4)^

Multiple contextual factors, including maternal education, socioeconomic status, community beliefs, feeding preferences, and knowledge of and exposure to mass media are determinants of malnutrition.^(5–7)^ Interventions that address these determinants, including social and behaviour change communication (SBCC), play a vital role in promoting optimal maternal as well as infant and young child feeding (IYCF) practices.^(8–10)^ SBCC interventions often generate awareness and in turn, improve knowledge to ultimately promote better practices; these interventions often also shift social norms which also shape behaviours. Specifically, mass media (such as radio, television, and newspaper) programmes have demonstrated positive effects on knowledge leading to better nutrition-related practices.^(11–13)^

Customised radio programmes have been successfully used to improve community awareness, as well as knowledge, attitudes, and practices on maternal, infant and young child nutrition in various parts of the world.^(14–16)^ For example, a two arm, quasi-experimental evaluation of a radio programme in northern Ghana found that after 12 months of exposure to a customised radio programme, the prevalence of both minimum dietary diversity and minimum acceptable diet among children 6 to 36 months improved significantly in the intervention group versus the comparison group (by 10 and 12 percentage points respectively); scores for maternal nutrition-related knowledge, attitudes and practices were also significantly higher in intervention communities than in comparison communities.^(15)^ A study in Ghana found that a radio campaign improved mothers’ health- and nutrition-related attitudes and increased children’s minimum acceptable diet in intervention areas more than in control areas (DID: 16.1 percentage points).^(17)^ A prior study in Nepal also found that children 6–24 months of mothers who had been exposed to *Bhanchhin Aama* were nearly 1.4 times more likely to meet minimum dietary diversity than those from mothers who were not exposed.^(18)^

Nepal’s multi-sectoral nutrition plan (MSNP II, 2018–2022) identified behaviour change as a key pathway to improving maternal, child, and adolescent nutrition.^(19)^ USAID’s *Suaahara* “Good Nutrition” Programme (2011–2023) was designed to support Nepal’s MSNP to improve the nutritional status of women and children in 42 of Nepal’s 77 districts. To achieve this goal, in 389 of Nepal’s 753 municipalities *Suaahara* interventions spanned nutrition, health, family planning, agriculture, water, sanitation, and hygiene (WASH), and nutrition governance. A wide range of SBCC interventions, including interpersonal communication (e.g. home visits), community events (e.g. mothers’ group meetings) and mass media (e.g. *Bhanchhin Aama* (Mother Knows Best)) were delivered to generate demand for and access to services and to motivate households to adopt optimal nutrition-related practices. Specifically, *Suaahara* SBCC activities promoted ten priority nutrition-related behaviours: participation in at least four antenatal care (ANC) checkups during pregnancy, consumption of at least 180 iron folic acid (IFA) tablets during pregnancy, adoption of a modern method of family planning (condom, pills, intra-uterine device, injectables or implants, or sterilisation), giving oral rehydration salt (ORS) and zinc for the treatment of young child diarrhoea, maternal dietary diversity especially consumption of eggs and animal source foods, child dietary diversity especially consumption of eggs and animal source foods, feeding more than usual to a child during and after illness, exclusively breastfeeding a baby from birth to six months, treatment of drinking water with an ideal practice (boiling, filtering, solar disinfection, or chlorination), and having soap and water available at the primary handwashing station at in or around the home.

To promote these ten behaviours, the *Bhanchhin Aama* radio programme began in 2013 as a weekly serial drama broadcast via a national radio network and in over 40 local FM stations of *Suaahara* intervention communities to encourage household discussions on these important topics and adoption of the promoted ideal practices.^(18,20)^ The serial drama included voices from the communities, field reports, interviews, and more. In 2016, during *Suaahara* II, *Bhanchhin Aama* continued broadcasting via the national radio network and increased to 125 local FM stations in the *Suaahara* intervention communities. In August 2018, *Suaahara* II-led capacity building efforts enabled a shift to production and broadcasting by local FM radio stations; networks of local FM stations covering about three to five districts within the same milieu of dialects and culture would collaborate and simultaneously broadcast to their communities. At the same time, a live call-in component was embedded so that audience queries and concerns could be addressed promptly, in locally relevant ways. *Bhanchhin Aama* was designed such that other *Suaahara* SBCC interventions could be complementary and they would all reinforce each other to remind and encourage adoption of ideal practices. *Bhanchhin Aama* also involved community leaders such as Nepal’s large cadre of female community health volunteers and health workers in the call-in segments, voices from the field (vox-pop) and reports of best practices and allowed listeners to seek answer to their submitted questions from their own local health service providers; all of this was part of creating an enabling environment that went beyond the *Bhanchhin Aama* episodes. Furthermore, to respond to changing media preferences of young women and men linked with increasing access to smartphones and the internet, *Suaahara* II also began sharing the *Bhanchhin Aama* episodes via Facebook, the most popular social media platform throughout rural Nepal; this was also a means of making this intervention available even to family members who had migrated. To increase interaction and audience participation in the programme, Facebook chat was introduced with a confirmed response time of 24 hours. Although *Suaahara* II’s focus was coverage of all communities in 42 districts, territorial borders could not restrict radio, social media, and online channels and thus the *Bhanchhin Aama* campaign had the potential to reach households across all of Nepal.

This paper assesses associations between maternal listenership to *Bhanchhin Aama* and *Suaahara* promoted maternal and child nutrition-related practices in Nepal.

## Methods

We used the Nepal Demographic Health Survey (NDHS) 2022, the most recent nationally representative cross-sectional household survey (https://dhsprogram.com/data). The dataset included information on socio-demographic characteristics and some of the ten-key nutrition-related behaviours promoted by *Bhanchhin Aama*. For analyses, we limited the sample to women having a child 0–23.9 months (*n* = 1,933). We further limited the sample for specific outcome indicators, as appropriate: only among mothers of children 6–23.9 months (*n* = 1,365) for child dietary diversity and only among mothers of children 0–5.9 months (*n* = 527) for EBF.

Primary outcome variables were selected based on prioritisation of topics from *Bhanchhin Aama* episodes during the two years prior to data collection, excluding WASH practices (since WASH-related behaviours were also being promoted by many development partners during this covid-19 pandemic time), continued breastfeeding at two years due to nearly 100% adoption of this practice, and those with a sample size of less than 300 (feeding more to a sick child; giving ORS and Zinc for young child diarrhoea and introduction of complementary foods to children between 6 and 8.9 months) which would limit power to detect an association.(22) Thus, the following five maternal and child nutrition-related practices were selected as the primary outcome variables:

1. Maternal minimum dietary diversity (MDD): whether the mother of a child under 2 years consumed food from at least five of ten food groups—grain, roots, tubers and plantains; pulses; nuts and seeds; dairy; meat, poultry and fish; eggs; dark green leafy vegetables; vitamin-A-rich fruits and vegetables; other vegetables; and other fruits—in the 24 hours prior to the survey^(21)^;
2. Modern method of family planning (MFP): whether the mother of a child under 2 years currently uses any MFP such as condoms, pills, intra-uterine devices, injectables or implants, or sterilisation to space or limit births;
3. Exclusive breastfeeding (EBF): whether the mother of a child less than 6 months only feeds breast milk and not any other foods or liquids to the child except medicines, vitamins, oral rehydration solution and minerals^(22)^;
4. Child MDD: whether a child (6–23.9 months) consumed food from at least five of eight food groups—grains, tubers, roots, and plantains; nuts and legumes; dairy; meat and fish; eggs; vitamin-A-rich vegetables and fruits; other vegetables and fruits; and breastmilk—in the 24 hours prior to the survey^(22)^; and
5. Growth monitoring and promotion (GMP): whether a child (0–23.9 months) was taken to the health facility to be weighed at least once in the three months prior to the survey.

Our primary exposure variable was *Bhanchhin Aama* listenership based on whether the woman reported having listened to *Bhanchhin Aama* at least once in the last three months.

Based on local context and prior studies, the following potentially confounding factors were included in the models: household wealth, agro-ecological zone of residency, caste/ethnicity, gender of the household head, family size, maternal age and maternal years of schooling, child age and child sex.^(23)^ Household wealth was measured based on the ownership of assets, household characteristics and access to services using principal components analysis and categorised into five different quintiles from lowest to highest.^(23)^ The caste/ethnicity variable was classified into four groups: *Brahmin*/*Chhetri, Janajatis*, *Dalits*, and others. Gender of the household head was defined as male or female. Family size was categorised as less than five members and five members or more. Maternal schooling was categorised into no schooling, 1–5 years of schooling, 6–9 years of schooling, and 10 or more years of schooling. Agro-ecological zone was based on whether the district has been classified by the government as mountain, hill or *terai* (lowland plains).

Adjusted logistic regression models were used to explore associations between maternal exposure to *Bhanchhin Aama* and the five outcomes. We applied sample weights, provided in the NDHS dataset that were calculated based on adjusting for non-responses.^(23)^ We used STATA Version 14^(24)^ for all statistical analyses.

The NDHS received ethical approval from Nepal Health Research Council as well as ICF Institutional Review Board and is a publicly available dataset (https://dhsprogram.com/). Separate ethical approval for these secondary analyses was not required.

## Results

Among the 1,933 mothers with a child less than 2 years in these analyses, more than two-fifths (45%) were from households in the poorest two wealth quintiles and nearly one-third (31%) belonged to the *Janajati* caste. More than two-thirds of the households sampled were male-headed (71%) and nearly three-quarters (73%) of households had five or more family members. More than one-third of mothers (36%) had either no formal schooling or only 1–5 years of schooling, whereas more than one-third also had completed 10 or more years of schooling. Approximately 16% of respondents listened to the *Bhanchhin Aama* radio programme at least once in the past three months. Nearly half of mothers (49%) and children (48%) aged 6–23 months met the standard for MDD. Not even one-third of mothers (31%) used MFP. More than half (57%) of mothers exclusively breastfed their children until the age of 6 months and more than half (61%) participated in GMP at least once in the 3 months prior to the survey (Table 1).

**Table 1.** Background characteristics, primary exposure and outcomes among mothers with a child under 2 years

	**%; Mean (SD)**
	***n*** **= 1933**
**Household size**	
Less than 5	26.9
5 and above	73.1
**Household caste/ethnicity**	
Brahmin/Chhetri	26.3
Janajati	30.6
Dalit	18.5
Other	24.6
**Household head gender**	
Male	71.0
Female	29.0
**Household wealth quintiles**	
Lowest	22.3
Second	22.3
Middle	19.7
Fourth	20.0
Highest	15.7
**Household agro-ecological zone of residency**	
Mountain	6.7
Hill	33.0
*Terai*	60.3
**Child age**	
0–5.9 months	27.8
6–11.9 months	22.9
12–23.9 months	49.3
Child age in months	11.1 (6.9)
**Child sex**	
Male	52.6
Female	47.4
**Maternal age**	
15–24 years	50.8
25–29 years	29.5
30 years and above	19.8
Maternal age in years	25.2 (5.1)
**Maternal schooling (in years)**	
None	19.0
1–5 years	16.6
6–9 years	29.7
10 and above years	34.7
**Mass media exposure**	
Listened to *Bhanchhin Aama* radio programme	16.4
**Outcomes**	
Maternal minimum dietary diversity	49.1
Modern method of family planning	29.4
Exclusive breastfeeding up to six months (*n* = 527)	56.6
Child (6–23.9m) minimum dietary diversity (*N* = 1365)	48.2
Child weight measured in past 3 months (*N* = 1892)	61.3

Compared to mothers unexposed to *Bhanchhin Aama*, more of those who were exposed to the programme met MDD (61% vs. 47%; *p* < 0.001) and used MFP (35% vs. 28%; *p* < 0.001). When comparing children whose mothers were exposed to *Bhanchhin Aama* versus those whose mothers were not, there was no difference in the proportion of children under 6 months who were exclusively breastfed, but a greater proportion of those 6–23 months met Child MDD (64% vs. 45%; *p* < 0.001). Likewise, participation in GMP was much more common for children of mothers who had listened to *Bhanchhin Aama* versus those who had not (81% vs. 58%; *p* < 0.001) (Table 2).

**Table 2.** Maternal and child nutrition outcomes, by exposure to Bhanchhin Aama and socio-demographic characteristics

	Maternal nutrition					Child nutrition								
		Maternal minimum dietary diversity (*N* = 1933)		Used modern method of family planning (*N* = 1933)		Exclusive breastfeeding up to six months (*N* = 527)			Child minimum dietary diversity (*N* = 1365)			Child participation in growth monitoring and promotion (*N* = 1892)		
	N	%	*P* value	%	*P* value	N	%	*P* value	N	%	*P* value	N	%	*P* value
**Listened to *Bhanchhin Aama in last three months***														
No	1,615	46.8	< 0.001	28.2	0.013	438	57.4	0.498	1144	45.2	< 0.001	1,582	57.5	< 0.001
Yes	318	60.8		35.4		89	52.8		221	64.2		310	80.9	
**Household size**														
less than 5	521	47.5	0.423	29.5	0.976	120	53.1	0.453	383	54.1	0.018	503	63	0.491
5 and above	1,412	49.7		29.4		407	57.6		982	46.0		1,389	60.8	
**Household caste/ethnicity**														
Brahmin/Chhetri	508	63.2	< 0.001	29.3	0.001	134	55.8	0.639	367	60.4	< 0.001	500	75.9	< 0.001
Janajati	591	50.8		36.1		175	52.5		407	51.4		582	67.9	
Dalit	359	37.3		26.5		88	60.2		262	39.0		350	57.1	
Other	475	40.9		23.4		130	60.6		329	38.1		460	40.4	
**Household head gender**														
Male	1,373	51.3	0.014	32.8	< 0.001	358	54.9	0.313	985	47.8	0.590	1,343	61.5	0.723
Female	560	43.8		21.1		169	60.3		380	49.5		549	60.9	
**Household wealth quintile**														
Lowest	431	29.8	< 0.001	33.5	0.248	99	64.1	0.134	321	39.1	< 0.001	420	63.6	0.007
Second	432	40.1		29.3		117	58.2		300	43.2		417	56.2	
Middle	381	45.5		24.4		106	60.5		267	45.4		373	54.8	
Fourth	386	59.5		29.8		107	55.4		271	53.3		379	62.7	
Highest	303	80.8		29.7		98	44.1		206	67.0		303	71.7	
**Household agro-ecological region of residency**														
Mountain	129	48.5	0.033	35.4	0.001	33	51.1	0.383	93	51.2	0.020	126	77.7	< 0.001
Hill	639	54.8		34.5		166	61.9		460	53.7		626	74.3	
Terai	1,165	46.1		25.9		328	54.5		812	44.8		1,140	52.5	
**Child age**														
Less than 6 months												527	71.6	< 0.001
6–11 months									432	32.4	< 0.001	432	63.8	
12–23 months									933	55.6		933	54.4	
**Child sex**														
Male						265	53.7	0.307	730	47.6	0.608	995	63.9	0.124
Female						262	59.5		635	49.0		897	58.5	
**Mother age**														
15–24 years	981	47.1	0.013	26.3	0.020	285	59.5	0.084	673	46.6	0.550	958	59.2	0.153
25–29 years	570	47.4		31.3		151	58.7		407	49.1		558	61.7	
30 years and above	382	56.8		34.7		91	44.0		285	50.9		376	66.3	
**Mother years of schooling**														
None	368	26.1	< 0.001	26.4	0.208	81	60.8	0.019	271	25.5	< 0.001	353	39	< 0.001
1–5 years	321	41.6		30.2		87	57.4		226	41.8		313	54.9	
6–9 years	574	45.5		32.8		156	65.5		409	48.8		564	64.4	
10 and above years	670	68.4		27.8		203	47.7		459	64.3		662	73.7	
**Total**	1,933	49.1		29.4		527	56.6		1,365	48.2		1,892	61.3	

Household size was not a differentiating factor for maternal or child outcomes. Differences by sex of the household head were seen for both maternal outcomes (but none for the child outcomes). Household caste and agro-ecological zone of residency were both differentiating factors for all outcomes other than EBF. Wealth differentiated those practising both maternal and child dietary diversity as well as GMP participation, but not EBF or use of modern family planning. Child outcomes varied by child age, but not child sex; maternal outcomes did not vary by either. On the other hand, maternal outcomes varied by maternal age and child outcomes did not. Maternal education, however, was an important factor for all outcomes, other than use of modern family planning (Table 2).

In final models, maternal exposure to *Bhanchhin Aama* was associated with 67% higher odds of mothers meeting MDD (OR: 1.67; *p* < 0.001; CI: 1.26–2.21), but not with maternal use of MFP methods. Maternal exposure to *Bhanchhin Aama* was not associated with EBF but was associated with 70% higher odds of children 6 to 23 months meeting MDD (OR: 1.70; *p* < 0.001; CI: 1.18–2.45) and 83% higher odds of a child participating in GMP (OR: 1.83; *p* < 0.001; CI: 1.28–2.63) (Table 3).

**Table 3.** Maternal listenership to *Bhanchhin Aama* and nutrition-related practices in Nepal

	Maternal minimum dietary diversity(*N* = 1933)	Used a modern method of family planning(*N* = 1933)	Exclusive breastfeeding up to six months(*N* = 527)	Child minimum dietary diversity (*n* = 1365)	Child participatio*n* in growth monitoring and promotion (*N* = 1892)
	OR, *P*, CI	OR, *P*, CI	OR, *P*, CI	OR, *P*, CI	OR, *P*, CI
**Maternal listenership to *Bhanchhin Aama in last three months***	1.67*** [1.26–2.21]	1.24 [0.92–1.67]	0.77 [0.44–1.35]	1.70** [1.18–2.45]	1.83** [1.28–2.63]
**Household size**					
less than 5	1				
5 and above	1.12 [0.86–1.45]	0.89 [0.68–1.17]	1.29 [0.79–2.09]	0.84 [0.63–1.12]	1.06 [0.80–1.41]
**Household caste/ethnicity**					
Brahmin/Chhetri	1	1	1	1	1
Janajati	0.65** [0.46–0.90]	1.49* [1.06–2.10]	0.73 [0.43–1.27]	0.87 [0.60–1.25]	0.86 [0.62–1.19]
Dalit	0.75 [0.52–1.10]	1.07 [0.72–1.58]	0.93 [0.42–2.05]	0.69 [0.46–1.04]	0.82 [0.56–1.20]
Other	0.69 [0.44–1.10]	1.44 [0.89–2.31]	1.18 [0.55–2.53]	0.59* [0.37–0.93]	0.47** [0.30–0.72]
**Household head gender**					
Male	1	1	1	1	1
Female	0.82 [0.63–1.08]	0.49*** [0.37–0.66]	1.19 [0.77–1.83]	1.07 [0.80–1.42]	0.94 [0.74–1.21]
**Household wealth quintile**					
Lowest	1	1	1	1	1
Second	2.04*** [1.46–2.84]	1.04 [0.73–1.47]	0.85 [0.44–1.62]	1.37 [0.89–2.11]	1.05 [0.73–1.51]
Middle	2.73*** [1.75–4.27]	0.80 [0.53–1.22]	1.05 [0.49–2.24]	1.47 [0.90–2.43]	1.05 [0.69–1.60]
Fourth	4.35*** [2.70–7.00]	0.96 [0.61–1.53]	0.84 [0.39–1.80]	1.88*** [1.16–3.04]	1.39 [0.88–2.19]
Highest	9.01*** [5.35–15.17]	0.99 [0.56–1.73]	0.65 [0.29–1.44]	2.49*** [1.45–4.28]	1.34 [0.83–2.16]
**Household agro-ecological zone of residency**					
Mountain	1	1	1	1	1
Hill	0.92 [0.63–1.37]	1.10 [0.71–1.69]	1.53 [0.64–3.68]	0.95 [0.57–1.56]	0.73 [0.45–1.21]
Terai	0.64 [0.40–1.02]	1.03 [0.61–1.76]	0.88 [0.32–2.42]	0.92 [0.52–1.60]	0.45** [0.26–0.77]
**Child age**					
Less than 6 months					1
6–11 months				1	0.61** [0.44–0.85]
12–23 months				3.41*** [2.55–4.56]	0.46*** [0.35–0.62]
**Child sex**					
Male			1	1	1
Female			1.20 [0.77–1.87]	1.05 [0.82–1.36]	0.85 [0.67–1.09]
**Maternal age**					
15–24	1	1	1	1	1
25–29	0.81 [0.62–1.06]	0.89 [0.67–1.19]	1.07 [0.65–1.74]	0.99 [0.73–1.34]	1.12 [0.87–1.45]
30 and above	1.00 [0.70–1.42]	0.81 [0.55–1.18]	0.66 [0.35–1.23]	1.12 [0.76–1.66]	1.23 [0.87–1.74]
**Maternal schooling**					
No schooling	1	1	1	1	1
1–5 years schooling	1.46 [0.97–2.20]	1.01 [0.68–1.50]	0.87 [0.43–1.73]	1.90** [1.22–2.96]	1.47 [0.98–2.21]
6–9 years schooling	1.30 [0.87–1.94]	1.27 [0.86–1.88]	1.31 [0.67–2.55]	2.16*** [1.41–3.32]	2.00*** [1.39–2.87]
10 and above years of schooling	2.19*** [1.40–3.41]	1.04 [0.68–1.59]	0.71 [0.35–1.45]	3.60*** [2.27–5.71]	2.40*** [1.60–3.59]

* *p* < 0.05; **p < 0.01; ***p < 0.001.

## Discussion

This study examined how maternal listenership to a customised radio programme was associated with promoted nutrition-related practices in Nepal, using data from a nationally representative survey. Positive associations were found for several behaviours prioritised by the programme: maternal and child dietary diversity and participation in GMP. Associations were not found, however, for other behaviours prioritised by the programme: use of modern family planning and EBF.

Our finding that maternal exposure to *Bhanchhin Aama* had a positive association with both maternal and child dietary diversity, is aligned with other studies from Nepal and other parts of the world.^(11–16)^ Using the most recent national dataset, these findings are consistent with a prior study that used *Suaahara* programme monitoring data from 2017 and found a positive association between *Bhanchhin Aama* listenership and child dietary diversity among children 6–23.9 months (OR 1.38; CI: 1.01–1.88) in intervention areas.^(18)^ Likewise, a secondary analysis of the 2016 NDHS data found that mothers exposed to health and nutrition programmes on the radio or television were 1.6 times more likely to feed their children a minimum acceptable diet.^(25)^ In Ethiopia, mothers of children 6–23 months exposed to mass media such as radio listenership, television watching or newspaper reading were nearly 4 times more likely to be knowledgeable about dietary diversification and over 8 times more likely to feed their children with nutrients from diverse food groups.^(26)^

The number of years of schooling of the mother and household wealth were significant predictors of both maternal and child dietary diversity in our study and across other LMICs.^(27,28)^ Better dietary practices require resources to purchase nutrient-rich foods, for example, and diversify diets. Similarly, better dietary practices among mothers with formal education reflects those key practices promoted on radio programmes may require cognitive skills to digest and implement what is being promoted; more educated women may also have greater agency or empowerment within their household to more easily be apply to apply these practices in their homes. The findings indicate that the customised radio programme alone cannot overcome structural inequalities rooted in socio-cultural practices. Disadvantaged households, particularly those with poorer and less-educated mothers, often lack the resources to act on recommendations. Therefore, SBCC programming, such as mass media interventions, likely need to be complemented with income-generating activities, for example, to ensure effectiveness across all groups and reduce nutrition-related inequities.

Maternal exposure to *Bhanchhin Aama* was not a significant predictor of whether the child less than six months was exclusively breastfed. This may be due to a smaller sample size and thus limited statistical power of the study. In Nepal, however, EBF practices are declining, as noted in the NDHS 2022. Exclusive breastfeeding is shaped by multiple interrelated factors; in this context, poverty, maternal employment, coverage of antenatal services, maternal workload, socio-cultural norms, and perceptions of insufficient breast milk have all been found to play a role.^(29,30)^ Therefore, exposure to SBCC promoting EBF may be insufficient to overcome these constraints. Further studies are needed to understand how to increase the rates of EBF throughout the country.

While maternal exposure to *Bhanchhin Aama* was not associated with the use of modern family planning, other factors emerged as important in this study: belonging to an upper caste family (*Brahmin/Chhetri*) increased the likelihood, whereas being from a female-headed household decreased the likelihood of using MFP. In Nepal, socio-cultural norms, gendered roles and responsibilities, myths and misconceptions about family planning methods, fear of side effects, and unequal decision-making power are key barriers to contraceptive use.^(31,32)^ In addition, there are caste and ethnicity-related barriers, particularly among Muslims, Dalits, and Terai/Madheshi women.^(33)^ Exposure to the *Bhanchhin* Aama, therefore, may not have been sufficient to overcome these more deeply entrenched socio-cultural norms and beliefs. Future family planning programmes must give greater attention to caste- and gender-related socio-cultural norms to increase the adoption of modern methods

The finding that maternal exposure to *Bhanchhin Aama* was positively associated with the uptake of GMP is encouraging, given its importance for detecting poor child growth and development and also for delivering important other child health and nutrition counselling services.^(34)^ The likelihood of participation was weaker among those living in the *terai*, having already turned 1 year old, and with mothers with less formal education, which is consistent with a prior study that found multiple socio-economic and demographic factors to generate variation in GMP utilisation.^(34)^ Given both supply- and demand-side factors, it is vital that policies and programmes simultaneously work with health systems and households to ensure quality GMP service provision and uptake.

Like all studies, this one has a few limitations. First, there are challenges with accurate measurement of the exposure variable: it is self-reported, simply a binary of ever versus never not capturing intensity of exposure, and limited to listenership on the radio specifically due to the survey questionnaire despite the possibility of exposure via social media, for example. *Bhanchhin Aama* (i.e. Facebook). Second, MDD outcomes were calculated based on the 24–hour recall method which is only a snapshot and may not be an accurate depiction of actual dietary diversity and is subject to both random error lowering the precision and systematic errors reducing the accuracy at each stage in the measurement process.^(35)^ Third, this study did not capture non-*Suaahara* interventions or other *Suaahara* SBCC interventions, such as interpersonal communication or community-based services, which may also have influenced the outcomes. As these interventions were complementary and promoted each other, interpreting the effects solely for *Bhanchhin Aama* radio programme would miss the point of *Suaahara’s* integrated model. Fourth, as this study used a cross-sectional dataset, causality cannot be inferred and findings should be interpreted with caution. These findings, however, are from a nationally representative survey, which is helpful in understanding the full geographic context of Nepal and how coverage of a radio programme may go beyond intervention areas to have nationwide effects. The findings will be helpful for designing future low-cost SBCC interventions for improving nutrition in Nepal or other low- and middle-income countries.

Maternal and child undernutrition continues to be a serious public health concern in Nepal and in many other LMICs. This study demonstrated that well-designed, implemented and promoted radio programmes can support families in their adoption of ideal practices. The best practices and learnings of the approach can be replicated in geographies with similar socio-demographic conditions. Programme planners in Nepal and other LMICs can use evidence from this study to develop feasible and culturally sensitive mass media-based interventions to improve maternal and child health and nutrition behaviours.
